# On the effects of multimodal information integration in multitasking

**DOI:** 10.1038/s41598-017-04828-w

**Published:** 2017-07-07

**Authors:** Ann-Kathrin Stock, Krutika Gohil, René J. Huster, Christian Beste

**Affiliations:** 1 Cognitive Neurophysiology, Department of Child and Adolescent Psychiatry, Carl Gustav Carus Faculty of Medicine, TU Dresden, Germany; 2 Department of Psychology, University of Oslo, Oslo, Norway; 3 Experimental Neurobiology, National Institute of Mental Health, Klecany, Czech Republic

## Abstract

There have recently been considerable advances in our understanding of the neuronal mechanisms underlying multitasking, but the role of multimodal integration for this faculty has remained rather unclear. We examined this issue by comparing different modality combinations in a multitasking (stop-change) paradigm. In-depth neurophysiological analyses of event-related potentials (ERPs) were conducted to complement the obtained behavioral data. Specifically, we applied signal decomposition using second order blind identification (SOBI) to the multi-subject ERP data and source localization. We found that both general multimodal information integration and modality-specific aspects (potentially related to task difficulty) modulate behavioral performance and associated neurophysiological correlates. Simultaneous multimodal input generally increased early attentional processing of visual stimuli (i.e. P1 and N1 amplitudes) as well as measures of cognitive effort and conflict (i.e. central P3 amplitudes). Yet, tactile-visual input caused larger impairments in multitasking than audio-visual input. General aspects of multimodal information integration modulated the activity in the premotor cortex (BA 6) as well as different visual association areas concerned with the integration of visual information with input from other modalities (BA 19, BA 21, BA 37). On top of this, differences in the specific combination of modalities also affected performance and measures of conflict/effort originating in prefrontal regions (BA 6).

## Introduction

Dual- or multitasking is an important faculty that allows us to cope with situations in which we are confronted with several task demands that occur either simultaneously or in rapid succession. In recent years, neuroscience has made considerable advances in understanding the neuronal mechanisms underlying this important faculty. However, our knowledge on the neurophysiological mechanisms underlying multi-tasking is likely to be biased. The reason for this is that multi-tasking is often examined using paradigms (e.g. psychological refractory period (PRP) paradigms) involving speeded responses to different sensory modalities (e.g. visual and auditory stimuli)^1–3^. Response selection problems in “multitasking” situations may therefore at least partly reflect processes related to attentional shifts between sensory modalities^4, 5^ and/or the cognitive effort to integrate input from different modalities. Furthermore, recent results show that the efficacy of response selection is in fact modulated by the similarity of information between sensory modalities^4, 6–9^. This emphasizes findings suggesting that response selection mechanisms are especially affected by the combination of input modalities when response selection capacities become fully stretched or even overstrained (e.g. in case of simultaneous input)^10–12^. Yet, the system neurophysiological mechanisms (i.e. underlying dissociable cognitive sub-processes related to multimodal information processing and integration) have remained elusive. To examine these processes in “multitasking” situations, neurophysiological methods like event-related potentials (ERPs) in combination with source localization methods seem well-suited. The main reason for this is that they allow to examine functionally different cognitive sub-processes related to attentional selection^5, 13, 14^ and response selection mechanisms^5, 14, 15^ by quantifying the P1 and N1 as well as the P3 ERPs, respectively. Yet still, ERPs are usually composed of various amounts of signals from different concurrently active brain sites^16–18^. This is an inherent feature of ERPs, but may cause problems when being interested in the contribution of different sensory modalities to response selection processes. More specifically, the problem is that ERPs represent a mixture of different sources of information, only some of which might be relevant to multimodal information processing. To overcome this limitation and to dissociate the effects of multimodal integration from other response selection mechanisms during multitasking, we applied an exploratory second order blind identification (SOBI) to the multi-subject ERP data. This allows to estimate and separate the activity of different brain sources^16, 19^. To the best of our knowledge, this has not been done before. As there is currently no knowledge about the precise neurophysiological mechanisms and the architecture of the associated functional neuroanatomical network underlying effects of multisensory integration on multitasking, the SOBI analyses were conducted in an exploratory fashion.

**Figure 1:**
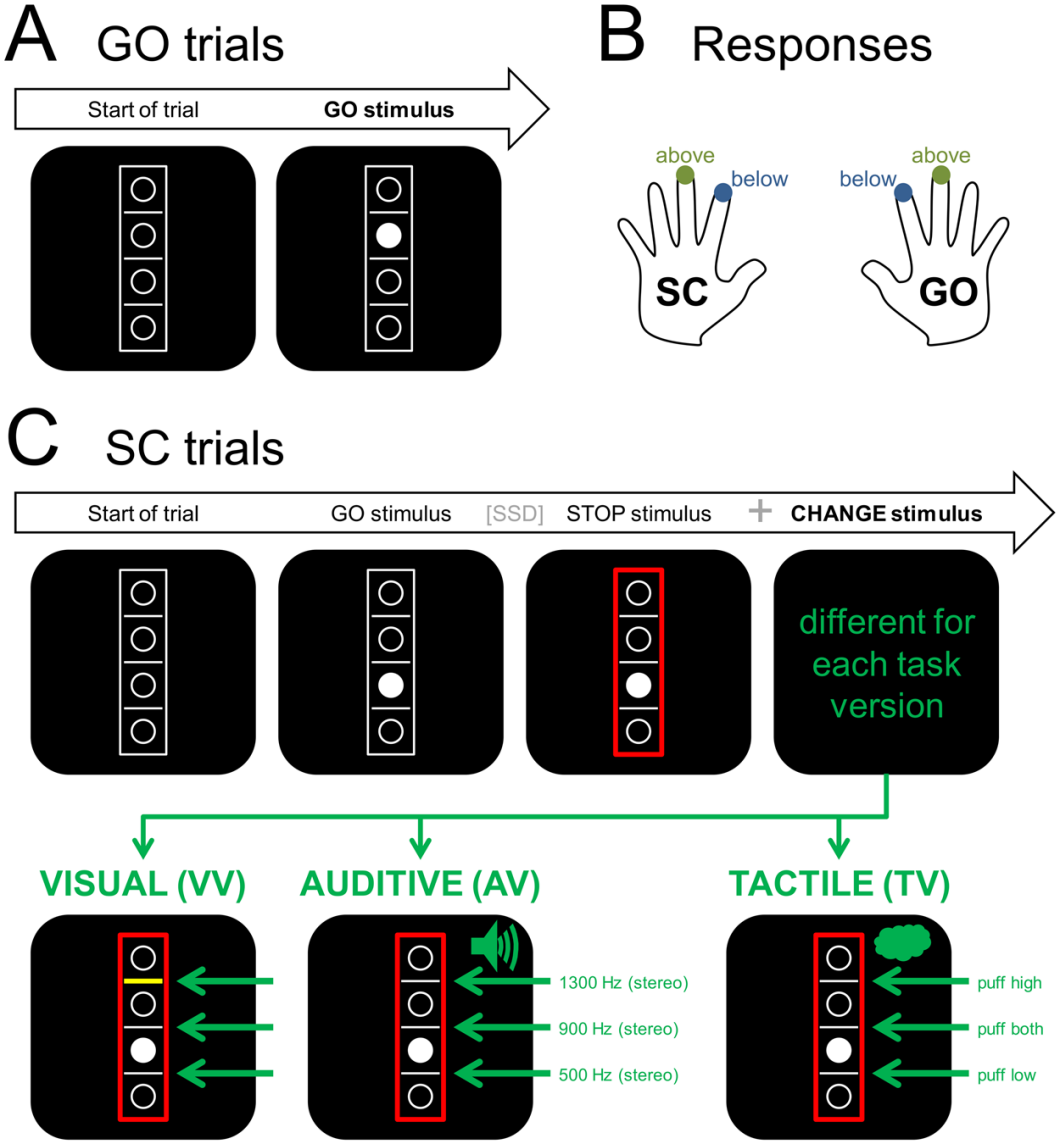
Illustration of the Stop change task and its variations. Despite all task version differences, the instructions were conceptually comparable and always required the subjects to judge the location of the target (white filled circle) in relation to either the middle reference line (GO trials) or to the reference line indicated by the CHANGE stimulus (SC trials). (**A**) The GO trial was identical for all task versions: After the empty visual array was presented for 250 ms, the target stimulus (white filled circle) appeared and stayed on the screen until the first given response. (**B**) Illustration of the required responses. The right hand should be used to respond in GO trials, while the left hand should be used in Stop-Change (SC) trials. To indicate that the target was located below the respective reference line, the index fingers had to be used. To indicate that the target was located above the respective reference line, the middle fingers had to be used. (**C**) Illustration of a SC trial. All SC trials started like regular GO trials, but after a variable stop signal delay (SSD), a stop signal (red frame) was introduced. The CHANGE signal was presented simultaneously. Participants always had to judge the target’s position *relative to* the reference line indicated by the CHANGE signal. In the visual condition (see bottom left), a yellow line would appear at one of the three line’s locations to assign a new reference line (visual stimulus illustrated for the upper line; green arrows point to the three possible positions). In the auditive task version, a binaural high (1300 Hz) tone coded for the high reference line, a medium tone (900 Hz) for the middle line and a low tone (500 Hz) for the low line. In the tactile task version, a high air puff (top of sternum) coded for the high reference line, a low puff (right below belly button) coded for the low line and a combination of both puffs coded for the middle line. Please note that the green symbols and illustrations were only added to facilitate the reader’s understanding but were not shown to the participants.

In the current study, we therefore set out to investigate this issue using ERP methods in combination with a SOBI-based signal decomposition used for source localization in a modified Stop-Change task (SCT)^20^ performed by healthy young adults. This task is essentially a combination of a PRP task and a stop-signal task. Put briefly, the task consists of simple visual Go trials which are followed by a STOP and CHANGE signal in 20% of all trials (see Fig. 1 and Methods section for illustration). The input modalities used to signal stopping and changing processes can be varied independently and systematically, which makes the paradigm suitable to examine the role of input modality in multitasking. While the STOP stimulus was always visual, the modality of the simultaneously presented CHANGE stimulus was varied (visual, auditive, or tactile) in an intra-individual design balanced for task version order. We hence compared a unimodal visual task version to two different bimodal task versions (audio-visual and tactile-visual). This helps to control for potentially special features of the involved modalities: Differences between the unimodal and the bimodal task versions should only be interpreted to reflect “true” multimodal information integration in case they do not dissociate between the two bimodal task versions. In contrast to this, any differences between the two bimodal task versions (or between all three task versions) should be attributed to modality-specific processing.

Concerning the neurophysiological measures, it has been suggested that visual P1 and N1 amplitudes are reduced in multimodal conditions because less resources are allocated to early visual stimulus processing when attention needs to be divided between several modalities^21–23^, especially when stimuli are “temporally aligned”^24^. As a recent study suggests that the requirement of multimodal information integration may enlarge the amplitude of the P3 component^4^, we also expected larger P3 amplitudes in the bimodal as compared to the unimodal task versions, probably reflecting increased cognitive effort^5, 14^. Given that all of our task versions are based on a visual array, we expected the SOBI to reveal effects of multimodal integration in visual association areas concerned with the integration of visual information and input from other modalities^25–27^. This hypothesis is also backed up by findings that multisensory input may activate many different cortical regions including sensory association areas in the temporal, parietal, and frontal lobes as well as primary sensory areas^28, 29^.

## Results

### Behavioral data

**Figure 2:**
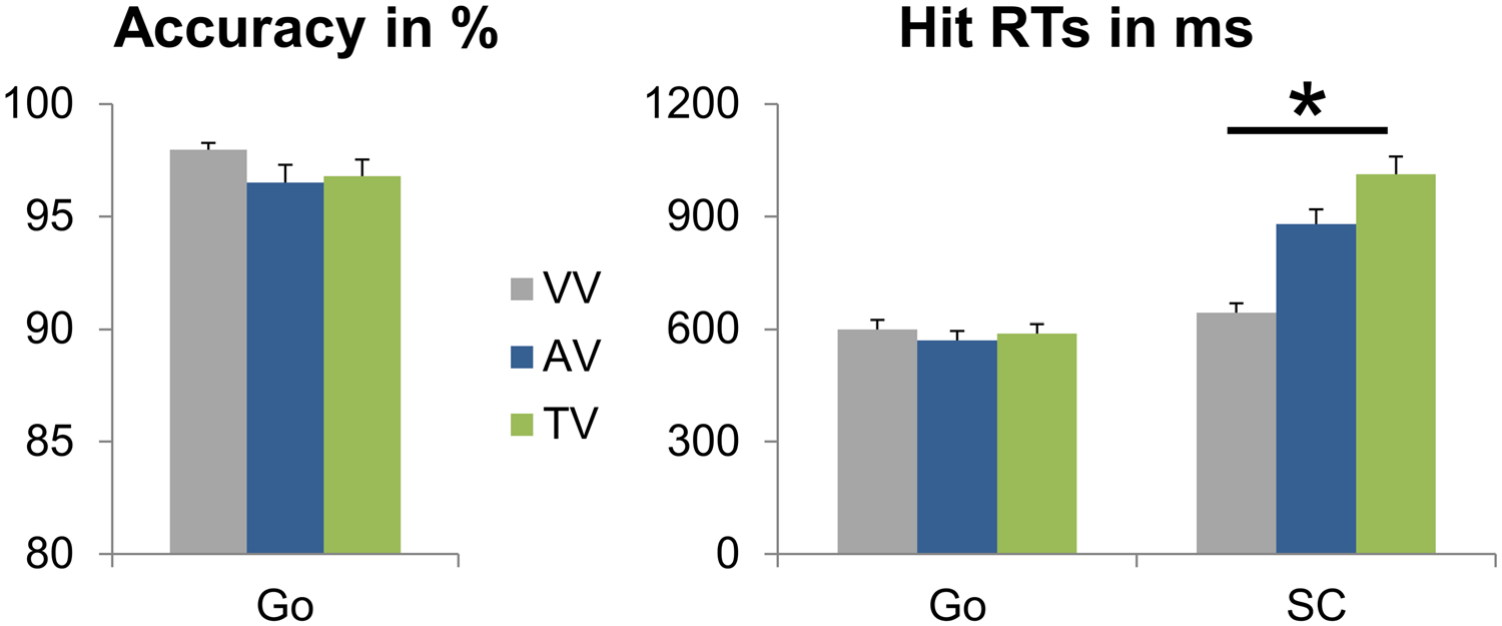
Task version differences in the behavioral data. VV = visual task version, AV = audio-visual task version, TV = tactile-visual task version. Please note that accuracy was not assessed for the SC condition, as the applied staircase procedure affects the accuracy in this condition.

The relevant behavioral data are depicted in Fig. 2. The analyses of simple Go trials revealed no differences between the three task versions for either accuracy (F(2,40) = 2.229; p = 0.133; partial η² = 0.100) or hit RTs (F(2,40) = 0.770; p = 0.466; partial η² = 0.037). Likewise, the stop-signal reaction time (SSRT)^5, 14^, which reflects the duration of the stopping process, did not show significant task version effects (F(2,40) = 0.889; p = 0.409; partial η² = 0.043; VV = 222.520 ± 11.480; AV = 221.892 ± 10.255; TV = 241.970 ± 16.535). The analysis of hit RTs in the SC condition revealed a main effect of task version (F(2,40) = 39.726; p < 0.001; partial η² = 0.665). Post-hoc t-tests showed that all three task versions significantly differed from each other (all p ≤ 0.001), with the unimodal VV version yielding the fastest SC hit RTs (643 ms ± 25), the bimodal AV version yielding intermediate SC hit RTs (880 ms ± 39) and the bimodal TV version yielding the slowest SC hit RTs (1012 ms ± 48). Due to the effects of the staircase procedure on accuracy (see methods section for detailed description and explanation), we refrained from analyzing the accuracy in SC trials.

In sum, we only found effects of task version in the SC condition. SC hit RTs differed between all three task versions, with the fastest correct responses in the unimodal VV version, followed by the bimodal AV version and the bimodal TV version. This means that despite playing an important role, multimodal integration was not the only factor modulating behavioral performance measures (in this case, there would have been no difference between the AV and TV task versions). Instead, there were also modality differences, which underlines the importance of how modalities are used/combined in multitasking paradigms.

### ERP results

**Figure 3:**
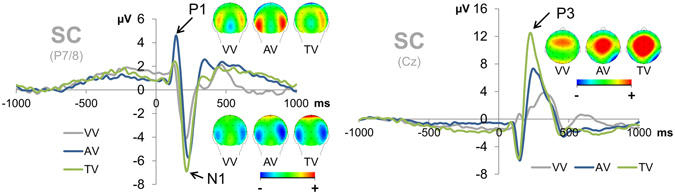
Event-related potentials. LEFT: Effects of task version on early attentional stimulus processing as reflected by the visual P1 and N1 components at electrodes P7 and P8. As can be seen, the amplitudes of the visually evoked P1 and N1 ERPs differed in case of simultaneous STOP-CHANGE stimulus presentation. While the P1 amplitude was increased only by the AV task version, the N1 was increased by both the AV and TV task versions, with the latter yielding the largest amplitude. VV = visual task version, AV = audio-visual task version, TV = tactile-visual task version. Topography maps are provided for each task and peak. RIGHT: Effects of task version on the central P3 component at electrode Cz. After simultaneous STOP-CHANGE stimulus presentation, the unimodal VV task version yielded the smallest P3 amplitudes, followed by the bimodal AV and TV task versions (with all three tasks significantly differing from each other). VV = visual task version, AV = audio-visual task version, TV = tactile-visual task version. Topography maps are provided for each task and peak.

Given that we only found modality (i.e. task version) effects in the SC condition, we limited our subsequent ERP analyses to this condition, thus leaving the simple Go condition out. We also only analyzed the STOP-related visual P1 and N1 components as the initial attentional processing of incoming stimuli may differ greatly with respect to both latency and amplitude in different primary and secondary sensory cortices. The visual STOP-related P1 and N1 ERPs are depicted in Fig. 3.

The analysis of the STOP-elicited visual P1 amplitudes yielded a main effect of task version (F(2,40) = 9.207; p = 0.001; partial η² = 0.315). Subsequent t-tests showed that the mean amplitude of the visual P1 was larger in the bimodal AV condition (4.151 µV ± 0.361) than in the unimodal VV condition (1.797 µV ± 0.496) (t(20) = −3.865; p = 0.001) as well as the bimodal TV condition (2.385 µV ± 0.524) (t(20) = 2.860; p = 0.010). Even though the amplitude values of the TV condition were intermediate, there was no significant difference between the VV and TV conditions (t(20) = −1.238; p = 0.230).

The analysis of the STOP-elicited visual N1 amplitudes yielded a main effect of task version (F(2,40) = 11.603; p = 0.001; partial η² = 0.367). Subsequent t-tests showed that there were significant differences between the N1 amplitudes of all task versions (all p ≤ 0.039). The unimodal VV task version yielded the smallest amplitudes (−3.089 µV ± 0.567), while the bimodal AV task version yielded intermediate N1 amplitudes (−5.089 µV ± 0.654) and N1 amplitudes were largest in the bimodal TV task version (−6.804 µV ± 0.914).

The central P3 peak elicited by the STOP+CHANGE stimuli in the SC condition is depicted in Fig. 3.

The analysis of the P3 peak amplitudes yielded a significant main effect of task version (F(2,40) = 18.032; p < 0.001; partial η² = 0.474). Post-hoc t-tests showed that there were significant differences between the P3 amplitudes of all task versions (all p ≤ 0.027). The unimodal VV task version yielded the smallest amplitudes (3.756 µV ± 0.861), while the bimodal AV task version yielded intermediate P3 amplitudes (7.217 µV ± 1.089) and P3 amplitudes were largest in the bimodal TV task version (12.377 µV ± 1.597).

In sum, we found that the different task versions (i.e. modalities) caused differences in the amplitude of all investigated ERPs elicited by/associated with the CHANGE stimuli presented in various modalities. This might reflect the increased task demands associated with simultaneously processing multiple input from different modalities. In response to this requirement, participants seem to have allocated more attentional resources to the processing of the visual stimulus (or perhaps the processing of all stimuli, as indicated by the increase in N1 amplitudes) and increased their effort (as indicated by the P3 amplitudes).

### SOBI results: Single trial-based signal decomposition reveals components and brain regions independently modulated by task modality and difficulty

Yet still, this does not explain why or from what aspect of stimulus processing these differences emerge. There are at least two factors which might contribute to the observed behavioral and neurophysiologic effects: modality-unspecific changes associated with multimodal information processing and modality-specific differences in information processing. In order to investigate these possibilities in greater detail, we performed an exploratory single trial-based signal decomposition to disentangle the underlying mechanisms accounting for the effects observed at the ERP level.

**Figure 4:**
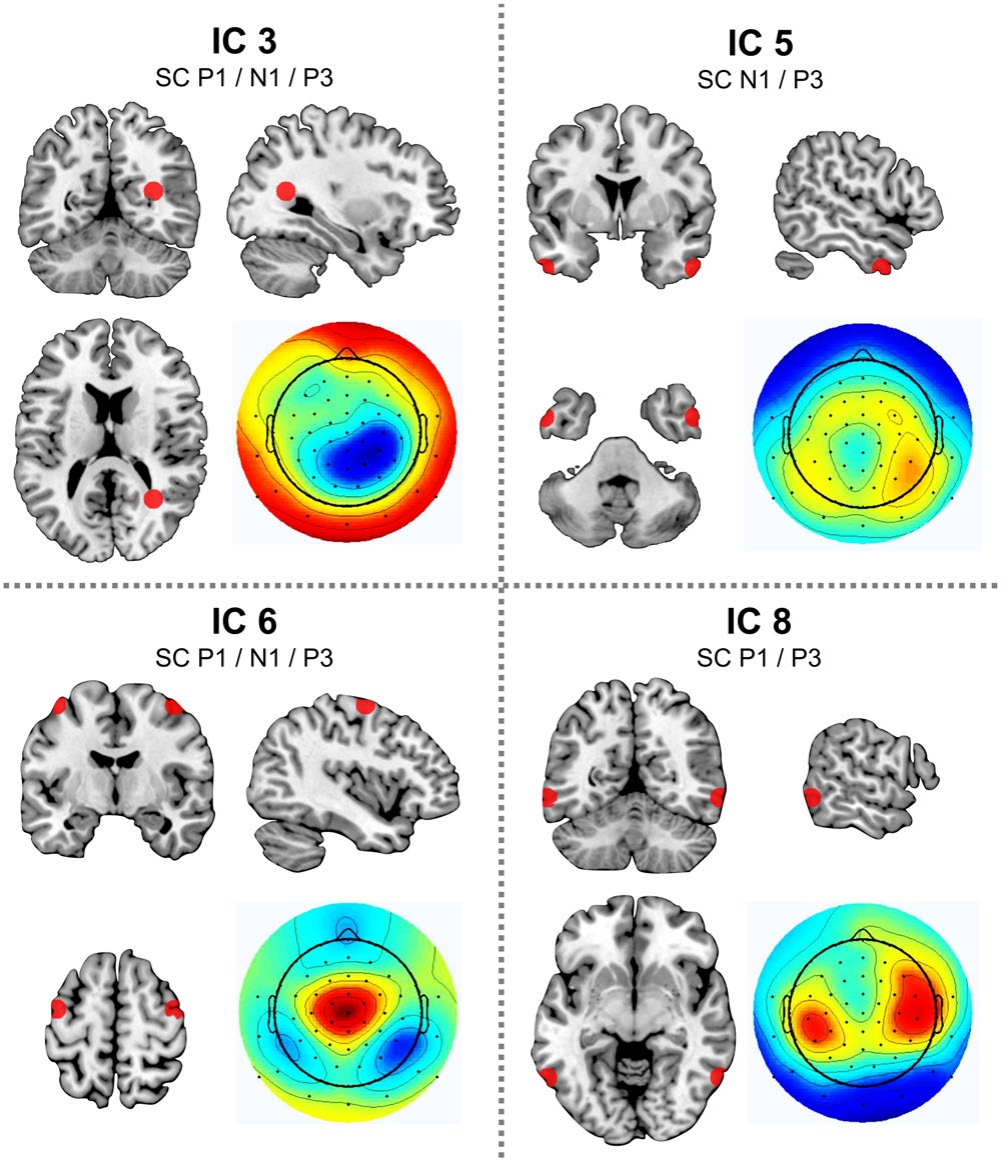
Relevant independent components. Illustration of the four independent components (ICs) which yielded significant task version differences within at least one of the time windows used for the quantification of ERP amplitude effects. IC 3 (top left) originated from BA 19 in the right middle temporal gyrus/occipital lobe (MNI coordinates: 31/−57/15) and yielded significant task version differences for all ERP time windows (i.e. P1, N1, and P3); the residual variance (RV) was 6.96%. IC 5 (top right) originated from bilateral BA 21 in the at the anterior part of the middle temporal gyrus (MNI coordinates: −53/53/3/−39) and yielded significant task version differences for two ERP time windows (i.e. N1 and P3); RV = 30.95%. IC 6 (bottom left) originated from bilateral BA 6 the middle frontal gyrus (MNI coordinates: −42/42/−6/62) and yielded significant task version differences for all ERP time windows (i.e. P1, N1, and P3); RV = 20.14%. IC 8 (bottom right) originated from BA 37 in the posterior parts of bilateral middle temporal gyrus (MNI coordinates: −62/62/−58/−7) and yielded significant task version differences in two of the ERP time windows (i.e. P1 and P3); RV = 10.22%.

To keep the analysis within a reasonable limit, we only analyzed the time windows which yielded significant task version differences at the level of ERP amplitudes (i.e. the time windows used for the quantification of P1, N1, and P3 components in the SC condition). Due to the fact that there were 10 ICs, we furthermore used a Bonferroni-correction, so that a significant difference was only assumed when p < 0.005. Altogether, four of the ten analyzed ICs reflected task version differences, but only two of those differed with respect to all investigated ERP time windows. To keep this text concise and easy to read, the results of all analyses are summed up in Table 1, while the text will be limited to the significant findings. The direction of effects revealed by subsequent post-hoc-tests will also be reported in the text, but the exact values obtained by averaging the ICs in the ERP quantification time windows will only be given in Table 2. ICs yielding significant task version differences are illustrated in Fig. 4.

**Table 1:** Overview of independent component analyses.

IC	SC P1			SC N1			SC P3		
	F	p	partial η²	F	p	partial η²	F	P	partial η²
1	0.494	0.590	0.024	00.677	0.506	0.033	10.649	0.212	0.076
2	0.711	0.478	0.034	00.884	0.382	0.042	20.698	0.098	0.119
3	**10.603**	**<** **0.001**	**0.346**	**13.851**	**<** **0.001**	**0.409**	**7.840**	**0.002**	**0.282**
4	1.068	0.336	0.051	1.196	0.305	0.056	1.144	0.327	0.054
5	1.314	0.280	0.062	**11.399**	**<** **0.001**	**0.363**	**15.951**	**<** **0.001**	**0.444**
6	**22.837**	**<** **0.001**	**0.533**	**38.855**	**<** **0.001**	**0.660**	**30.786**	**<** **0.001**	**0.606**
7	0.540	0.573	0.026	0.576	0.488	0.028	6.012	0.011	0.231
8	**20.487**	**<** **0.001**	**0.506**	2.547	0.107	0.113	**8.539**	**0.002**	**0.299**
9	1.181	0.317	0.056	2.470	0.112	0.110	1.109	0.339	0.053
10	0.185	0.815	0.009	2.216	0.135	0.100	0.437	0.646	0.021

In this table, we summarized the results of the repeated-measures ANOVAs for all ten ICs. Significant differences are marked with bold font. Please note that due to Bonferroni-correction, the significance threshold was lowered to p ≤ 0.005.

**Table 2:** Relevant independent components.

IC	SC P1				SC N1				SC P3			
	Post-hoc	VV	AV	TV	Post-hoc	VV	AV	TV	Post-hoc	VV	AV	TV
3	AV > (TV & VV)	−0.339 ± 0.062	0.213 ± 0.124	−0.381 ± 0.128	TV < (AV & VV)	−0.479 ± 0.169	−0.597 ± 0.186	−1.475 ± 0.269	TV < (AV & VV)	−0.838 ± 0.128	−0.725 ± 0.203	−1.516 ± 0.275
5	n.s.	n.s.	n.s.	n.s.	TV > (AV & VV)	−0.984 ± 0.132	−0.870 ± 0.154	−0.365 ± 0.186	VV > (AV &TV)	0.378 ± 0.103	−0.465 ± 0.126	−0.268 ± 0.187
6	VV > (AV & TV)	0.138 ± 0.058	−0.635 ± 0.100	−0.653 ± 0.139	TV > AV > VV	0.654 ± 0.085	1.043 ± 0.132	2.190 ± 0.238	TV > AV > VV	0.629 ± 0.147	1.316 ± 0.163	2.224 ± 0.245
8	AV < (VV & TV)	0.127 ± 0.055	−0.536 ± 0.114	0.076 ± 0.117	n.s.	n.s.	n.s.	n.s.	VV < (AV & TV)	0.198 ± 0.096	0.811 ± 0.142	0.730 ± 0.233

Significant results obtained by means of post-hoc t-tests and IC values averaged over the time windows which were used for ERP quantification (see Table 1). Values are only reported for analyses of ICs yielding significant task version effects (refer Table 1). VV = visual-visual task version, AV = audio-visual task version, TV = tactile-visual task version. In the Post-hoc columns, significant differences are indicated by the “ > ” and “<” signs while non-significant differences are indicated by the “&” sign and grouping the respective task versions within brackets.

IC 3 was the first component to show significant main effects of task version in all of the assessed ERP time windows (all F ≥ 7.840; p ≤ 0.002). Its topography exhibited a right centro-parietal maximum and inverse modeling indicated a global maximum in BA 19 in the right middle temporal gyrus/occipital lobe (MNI coordinates: 31/−57/15, see Fig. 4). Post-hoc t-tests revealed that in the P1 time window, the AV task version yielded lower activity than the VV and TV task versions (all p ≤ 0.001) while TV and VV did not differ from each other (t(20) = −0.260; p = 0.798). In the N1 and P3 time windows, TV showed increased activity compared to the VV and AV task versions (all p ≤ 0.012) while AV and VV did not differ from each other (all p ≥ 0.522).

IC 6 was the second component to show significant main effects of task version in all of the assessed ERP time windows (all F ≥ 20.421; p < 0.001). Its topography exhibited a central maximum with bilateral posterior temporal minima. Inverse modeling indicated a global maximum in BA 6 in the middle frontal gyrus (MNI coordinates: (−)42/−6/62, see Fig. 4). Post-hoc t-tests revealed that in the P1 time window, the unimodal VV task version yielded positive values while the bimodal AV and TV task versions yielded negative values (all p ≤ 0.001) while TV and AV did not differ from each other (t(20) = 0.112; p = 0.912). In the N1 and P3 time windows, all task versions significantly differed from each other (all p ≤ 0.002), with the TV task version yielding the largest positive values, followed by the AV version, and the VV version yielding the smallest positive values.

IC 5 showed significant main effects of task version at the N1 and P3 ERP time windows (all F ≥ 11.399; p < 0.001). Its topography showed that the underlying source most strongly projected to right parietal electrodes. The main source of activity was localized in BA 21 in the at the anterior part of the middle temporal gyrus (MNI coordinates: (−)53/3/−39, see Fig. 4). Post-hoc t-tests revealed that in the N1 time window, TV yielded less negative values than the VV and AV task versions (all p ≤ 0.001) while AV and VV did not differ from each other (t(20) = 0.811; p = 0.427). In the P3 time window, the unimodal VV task version yielded positive values while the bimodal AV and TV task versions yielded negative values (all p ≤ 0.002) and did not differ from each other (t(20) = 1.669; p = 0.111).

Last but not least, IC 8 showed significant main effects of task version in the P1 (F(2,40) = 20.487; p < 0.001; partial η² = 0.506) and P3 (F(2,40) = 8.539; p = 0.002; partial η² = 0.299) ERP time windows. Its topography showed that temporal electrodes on both sides were most strongly associated and the main source of activity was localized in BA 37 in the posterior parts of the middle temporal gyrus (MNI coordinates: (−)62/−58/−7, see Fig. 4). Post-hoc t-tests revealed that in the P1 time window, the AV task version yielded negative values while the VV and TV versions yielded positive values (all p < 0.001). The VV and TV versions did not differ from each other (t(20) = −0.466; p = 0.646). In the P3 time window, the unimodal VV task version yielded smaller positive values than the bimodal AV and TV task versions (all p ≤ 0.012), while AV and TV did not differ from each other (t(20) = −0.528; p = 0.603).

In sum, the effects of multimodal processing (i.e. the differences between by uni- vs. bimodal stimulus input) caused activation differences in BA 21, BA 37, and BA 6. In contrast to this, the modality differences observed for hit RTs as well as the N1 and P3 ERP amplitudes were only reflected by activity changes within and BA 6. Lastly, the P1 amplitude difference caused by the AV task version in the SC condition was reflected by BA 19 and BA 37.

## Discussion

This study investigated the notion that our current understanding of multitasking might be confounded with multimodal integration. As this has never been systematically assessed at the neurophysiological level, we set out to thoroughly investigate the effects of multimodal stimulus input on multitasking behavior as well as the underlying cognitive sub-processes and functional neuroanatomical networks using information theoretical approaches applied on neurophysiological data.

We found that multimodal information processing and integration (as defined by the differences between the unimodal and the bimodal task versions) clearly impaired multitasking as the unimodal visual task version yielded a better performance (i.e. faster responses) than the two bimodal task versions. We however also observed modality-specific RT differences as the tactile-visual task version yielded slower responses than the auditory-visual task. Given that the speed of signal transduction or effects of divided attention cannot provide a plausible explanation for the observed “order” of task version RTs^30^, we concluded that those additional RT effects were most likely due to modality-specific differences. These could simply be based on practice effects, but they could also be caused by the number of required “spatial information transformations”: In the unimodal VV task version, all stimuli were presented on the screen together so that no transformation needed to be made in order to spatially relate the target stimulus to the new reference line. By contrast, the bimodal AV task version required one spatial transformation as an abstract stimulus property (i.e. tone frequency) had to be related to a spatial position on the screen. Lastly, the bimodal TV task version possibly required two spatial transformations as the proprioceptive input from the tactile stimuli was first encoded in an egocentric space and then needed to be transferred to the allocentric reference system of the paradigm. Taken together, this allows for the conclusion that both multimodal information integration (indicated by differences between uni- and bimodal task versions) and modality-specific aspects of information processing (indicated by differences between all three task versions, especially the two bimodal ones) may confound measures of multicomponent behavior. Furthermore, we found no effects of task version on simple Go trials, which proves that the participants’ baseline performance was comparable across all three appointments and that the obtained effects are strictly confined to multicomponent behavior. Therefore, neurophysiological analyses were confined to the SC condition.

To investigate which cognitive sub-processes of multicomponent behavior were modulated by multimodal information processing, we quantified ERPs known to reflect early attentional visual processing (visual P1 and N1^13^) and response selection/cognitive effort (P3^5, 14^).

Previous studies suggested that multimodally divided attention yields smaller P1 and N1 amplitudes as attentional resources need to be “split up” between the involved primary and secondary sensory cortices^21–23^. However, we found larger P1 and N1 amplitudes in case of multimodal input. This contradictory finding might be explained by the fact that we did not ask the participants to perform two entirely unrelated tasks in response to simple stimulus input. As our task was based on a visual array which was only ‘complemented’ by input from other modalities, a decrease in visual information processing would have almost certainly been detrimental for task performance (i.e. the maintenance of high levels of visual attention is actually beneficial to task performance).

More specifically, we found that the initial bottom-up processing of visual information was enhanced by simultaneous audio-visual stimulus input, as indexed by enhanced visual P1 amplitudes. This finding can be explained by the fact that auditory information reaches the primary cortex much earlier than tactile or visual information^30^. It hence seems plausible that only auditive input may have reached the cortex with a sufficient temporal advantage to influence early attentional visual processing^13^ even at its earliest stages, while tactile input failed to have the same effect.

More importantly, however, we found that our behavioral findings were reflected by a modality effect on both the visual N1 and the central P3, which showed amplitude differences between all three task versions: For both ERPs, the unimodal visual task version yielded the smallest N1 and P3 amplitudes while the bimodal AV task version yielded intermediate amplitudes and the bimodal TV version yielded the largest amplitudes. The N1 is known to reflect attentional visual processing^13^, but it may also encompass early aspects of top-down guided task-related attention^31–34^. P3 amplitudes have been shown to reflect response control/cognitive effort as the amplitude of the central P3 often increases with task difficulty^5, 15, 35, 36^. In the context of this paradigm, larger P3 amplitudes have been associated with decreased behavioral performance and increased cognitive effort^5, 14^. It has furthermore been suggested that multimodal information integration may enlarge the amplitude of the P3 component^4^. As the N1 an P3 amplitudes clearly mirror the behavioral data, it seems that the participants tried to compensate modality-specific increases in task difficulty (think required spatial transformations) by allocating more attentional resources to task-related visual stimulus processing and investing more cognitive effort/control during the response selection stage of the bimodal tasks.

The clear differences between the two bimodal tasks suggest that the observed effects were at least partly driven by modality-specific factors and not just by modality-unspecific multimodal information integration. Yet, both may have similar effects on the required cognitive effort (both should always be lowest in the unimodal condition). Therefore, it cannot be entirely ruled out that modality-unspecific multimodal integration also played a role in these processes. In other words, the ERP analyses alone do not clarify in what way or proportion these two factors have contributed to the observed behavioral and ERP differences.

To answer these questions, we identified the mechanisms underlying the observed effects by decomposing the ERP signal. The analyses of the obtained ICs revealed that different components/brain regions accounted for the modality effects/task difficulty.

Of note, the modality-specific differences observed at both the behavioral and ERP level (i.e. TV > AV > VV) were reflected in both the N1 and P3 time windows, but only by one component. IC6 has a topography that is very similar to that of the P3 ERP (compare Figs 3 and 4) and the premotor cortex, which has been identified as the main source of IC6, is known to play a role in planning and executing complex motor responses. Furthermore, it is part of a spatial awareness network^25, 27^ involved in planning the strategy for navigating in attentional landscapes^25, 27, 37^. Within the context of this network, the dorsolateral premotor cortex has been suggested to be involved in (spatial) attentional selection including processes required for multimodal information integration^37–39^. It has furthermore been linked to intersensory attention^26, 38, 40^ and has been shown to play an important role in the processing of multimodal stimulus input^29, 38, 39^.

Based on these findings, it can be stated that modality variations caused differences in premotor cortex activation, which likely contributed to the observed slowing of responses and enlarging of the N1 and P3 amplitudes.

We however also found effects of multimodal integration as defined by the difference between uni- vs multimodal task versions (in the absence of significant differences between AV and TV). These were reflected in IC 5/BA 21, IC 6/BA 6, and IC 8/BA 37. BA 21 is part of the visual association cortex located in the middle and inferior temporal gyri^25^. These regions play a key role in multimodal visual processing and the alternation between the external and physical self/world, most likely based on the integration of visual, proprioceptive, tactile and vestibular modalities^25, 29, 38^. Like BA 21, BA 37 is also part of the visual association cortex and plays a key role in multimodal visual processing and the alternation between external and physical/proprioceptive information (which is strongly required in the TV task version), most likely based on the integration of visual, proprioceptive, tactile and vestibular modalities^25, 38, 41^.

This means that even though the modality-specific differences might have been more prominent at the behavioral and ERP level, multimodal information processing affected the activity in a larger number of cortical regions, encompassing both attentional and visual association areas. Matching the fact that our task was based on a primarily visual setup, we found that bimodal stimulus input altered the activity in several visual association cortices concerned with the integration of visual information with input from other modalities. Hence, it can be rather safely stated that both modality-specific differences as well as modality-unspecific multimodal integration affects behavior and its neurophysiological correlates. Yet, the effect of modality-specific differences on multitasking seemed to be confined to areas in the prefrontal cortex while a wider range of association areas was concerned with the general integration of input from several modalities.

Lastly, we found the enhanced bottom-up processing of visual information in the AV condition observed for the P1 amplitudes to be reflected by decreased activity in IC8/BA 37 and increased activity in IC 3/the right BA 19. T he right BA 19 is also a visual association area and part of the spatial awareness network proposed by Mesulam^37^. The inferior parietal lobule has been associated with spatial processing including the egocentric representation of one’s own body, which plays a role in the tactile-visual task version we used^25, 29, 39^. Posterior parietal cross-modal transition zones including BA 19 are also known to be involved in the integration of memory, vision, and proprioception. This brain region is also important for perceiving simultaneous sensory inputs^25, 29, 38^. As already argued above, the fact that auditory information reaches the primary cortex much earlier than tactile or visual information^30^ could explain why we find such effects for the AV condition, but not for the TV condition.

One aspect worth discussing is the temporal spacing of multimodal input. In previous studies using the same paradigm, we also employed a SC condition with a stimulus onset asynchrony (SOA) of 300 ms^5, 14^. This condition was left out in the current study because we felt it would further complicate matters without contributing to our main question. Yet still, it should be mentioned that effects of multimodal information integration should decrease as the duration of the SOA between the STOP and CHANGE stimuli increases. There are two reasons motivating this assumption: The first reason is that Verbruggen *et al*.^20^ have demonstrated that the task goal associated with the STOP stimulus is usually terminated within less than 300 ms so that SOAs of 300 ms or more should not necessitate the integration of STOP and CHANGE information in the same way as a simultaneous stimulus input. The reason for this assumption is that there seems to be a “temporal window of integration” only within which we are able to integrate input from different sensory modalities^42^. Hence, longer the SOAs may “push” the second stimulus out of that window and thus impede multimodal information integration.

Furthermore, it needs to be noted that our findings might also have implications in a clinical context. There are several neurological and psychiatric diseases, such as ADHD or schizophrenia, that have not only been associated with executive dysfunction, but also show differences and/or deficits in multisensory information integration. ADHD patients, for example, do not only show inattention, but also executive dysfunction and weaker inference control^43, 44^. Also, a recent resting-state fMRI-based study has shown that while the interconnectivity within primary cortices of ADHD patients is good, these regions show reduced functional connectivity to brain areas that are mainly concerned with attention regulation and executive processes^45^. Taken together, ADHD patients seem to have a less effective allocation of attention to different sensory inputs as well as the impaired connection of primary cortices to (pre)frontal brain areas. This suggests that some aspects of the executive deficits found in ADHD patients may to some degree be confounded or worsened by multimodal integration difficulties. Further supporting this view, a recent study has shown that under certain circumstances, “the ability to take contextual information into account during conflict monitoring is preserved in patients with ADHD despite this disorder being associated with changes in executive control functions overall”^46^. Something similar may hold true for schizophrenia. Despite all of the differences between those two disorders, patients with schizophrenia have also been shown to display modality-specific attention deficits^47^. Further underpinning this assumption, a recent fMRI study has suggested “a prominent role for dysfunction within auditory, sensorimotor and parietal areas relative to prefrontal CCN nodes during multisensory cognitive control”^48^. Taken together, this further supports the notion that at least some of the executive dysfunctions reported in several clinical research areas may have been influenced or aggravated by deficits in multisensory information integration.

Summing up our study, we found that multimodal stimulus presentation has profound, yet specific effects on behavioral measures and neurophysiological mechanisms during multitasking. We found that both general multimodal information integration and modality-specific aspects (potentially related to task difficulty) modulate behavioral performance and associated neurophysiological correlates. Tactile-visual input caused larger impairments in multitasking than audio-visual input. The ERP data shows that simultaneous multimodal input increases early attentional processing of visual stimuli (i.e. P1 and N1 amplitudes) as well as measures of cognitive effort and conflict (i.e. central P3 amplitudes). General aspects of multimodal information integration modulated the activity in the premotor cortex (BA 6) as well as different visual association areas concerned with the integration of visual information with input from other modalities (BA 19, BA 21, BA 37). On top of this, the specific combination of modalities also affects performance and measures of conflict/effort originating in prefrontal regions (BA 6). Hence, there are at least two factors which may potentially confound the inferences we draw from multitasking experiments that combine stimuli from different modalities.

## Methods

### Sample

The study included 24 participants 3 of whom had to be excluded due to drop-out between appointments. Thus, our final sample consisted of 21 healthy right-handed participants (14 females) aged 18 to 31 (mean age = 24.8, SD = 3.5). Due to our within-subjects design, each participant had to perform three different versions of our experimental paradigm at three separate appointments. All of these appointments had to be spaced by at least one and no more than seven days apart. A written informed consent form was signed by each participant before the experiment and each participant was reimbursed with 60 € after completing all three appointments. Subjects were treated in accordance with the declaration of Helsinki. The study was approved by the ethics committee of the Faculty of Medicine of the TU Dresden. All methods were performed in accordance with the relevant guidelines and regulations.

### General experimental paradigm

To dissociate the effects of multimodal stimulus input on multitasking in situations with simultaneous stimulus presentation, we used a modified version of the Stop-Change paradigm introduced by Verbruggen *et al*.^20^. While input modalities differed, the basic structure of the task was the same in all three investigated task versions:

At each of their three appointments, subjects were comfortably seated at a distance of 57 cm from a 17 inch CRT computer monitor in a sound-attenuated room. The participants were instructed to respond with the index and middle fingers of both hands using four different keys (“S”, “C”, “N”, and “K”) on a regular computer keyboard placed in front of them. “Presentation” software (Version 17.1 by Neurobehavioral Systems, Inc.) was used to present the stimuli, record the behavioral responses (response times and accuracy) and to synchronize with the EEG. Each task version of the paradigm consisted of a total of 720 trials, which were subdivided into 8 blocks, taking about 25 minutes from start to end. All employed versions of the task consisted of 576 simple “GO” trials 144 “Stop-Change” (SC) trials which were presented in a pseudorandomized order as explained in the following. All stimuli were presented on a black background. Each trial started with a central 250 ms presentation of an empty array. This array consisted of a white rectangle containing 4 vertically arranged white bordered circles separated by 3 white horizontal lines (see Fig. 1). In the “GO” condition, which constituted the majority of trials, one of the four circles was then filled with white color, thus becoming the target/GO stimulus. Participants were asked to indicate whether this stimulus was located above or below the middle (reference) line. In case the target was located above the middle white line, participants had to respond with their right middle finger (by pressing the “K” key). If the target was located below the middle line, participants had to respond with their right index finger (by pressing the “N” key) for the correct response. A speed up sign (the German word “Schneller!” which translates to “Faster!”) was presented above the stimulus array whenever participants did not respond within 1000 ms after the onset of the target. The array including the target and the speedup sign (whenever presented) remained on the screen until trial was ended by a button press.

The remaining 144 trials were so-called stop-change (SC) trials. Like GO trials, all SC trials started with a 250 ms presentation of the empty array, followed by a GO stimulus. After a variable stop-signal-delay, the GO stimulus was followed by a STOP stimulus (the border of the rectangle turned from white to red, see Fig. 1). Participants were required to inhibit/stop their right hand response to the GO stimulus as soon as the STOP stimulus was added to the visual array. Additionally, the STOP stimulus was always complemented by a simultaneous CHANGE signal which required participants to respond with their left hand. In short, the CHANGE stimulus coded for one of the three white lines in the visual array, thus “assigning” a new reference line. Our three task versions used visual, or auditory, or tactile CHANGE stimuli (for details, please see following text section) but the three lines were in effect equally often in all task versions. Irrespective of the CHANGE stimulus modality, participants were asked to spatially relate the target (white circle) to the newly assigned reference line. Whenever the target (which remained in the same location throughout the entire trial) was located above the newly indicated reference line, a left middle finger press of the “S” key was required. When the target was located below the newly assigned reference line, a left index finger press of the “C” key was required. In case of response times (RTs) longer than 2000 ms, the German word “Schneller!” (translates to “Faster!”) was presented above the box until the participant responded to end the trial. Of note, all kinds of trials were only included in the subsequent analyses when responses occurred with a delay of at least 100 ms after the onset of the respective stimulus (GO stimulus in GO trials and STOP+CHANGE stimulus in SC trials) and before the presentation of the speedup sign.

Lastly, the stop-signal delay (SSD) was dynamically and individually adjusted by means of a staircase procedure^20, 49^ after each SC trial. Initially, the SSD was set to 250 ms. Whenever the participant correctly responded during an SC trial (i.e. did not respond before the presentation of the CHANGE stimulus and correctly responded to the CHANGE stimulus described above), the SSD was decreased by 50 ms. Whenever the participant made an incorrect response to the CHANGE stimulus and/or responded before the presentation of the CHANGE stimulus, the SSD was increased by 50 ms. To keep the trial duration within reasonable limits, SSD variation was restricted to a range from 50 to 1000 ms. As a result, the staircase procedure approximately yields a 50% probability of successfully performed SC trials. As a consequence of this combination of criteria, the accuracy of responses of the CHANGE stimulus approximates 50% in SC trials. Hence, only the RTs of correct responses can be analyzed for SC trials.

### Visual, auditory and tactile task versions

To investigate the effects of multimodal information integration, we systematically varied the modalities in which the task-relevant CHANGE stimuli were presented and compared a unimodal visual task version to two different bimodal task versions. The CHANGE modality could either be visual, auditory, or tactile, as illustrated in Fig. 1. All three versions were completed by the participants in a counterbalanced order.

In the visual task version (VV), the CHANGE stimuli were bold yellow bars. In each SC trial, one of the three horizontal lines would turn into a thick yellow bar, thus becoming the new reference line. These remained on the screen until the participant responded to the CHANGE stimulus.

In the auditory task version (AV), the CHANGE stimulus was a 200 ms sine tone presented via headphones. There were 3 differently pitched tones (low/600 Hz, middle/900 Hz, and high/1200 Hz) presented at a 75 dB sound pressure level. The middle tone represented the middle reference line while the high and low tones stood for the high and low reference lines, respectively. Given that all tones were presented to the two ears via headphones, they did not have inherent spatial properties, as compared to the visual CHANGE stimuli.

In the tactile task version (TV), the CHANGE stimulus was a 100 ms air puff delivered to the sagittal line of the participant’s torso. As some participants were not very sensitive in this area, there were only 2 different locations where the air puff was delivered. One location was on the chest of the participant (at the top of the sternum), which represented the top reference line. The other location was right below the belly button of the participant and represented the bottom reference line. When both (top and bottom) locations where stimulated together, this coded for the middle reference line.

### EEG recording and preprocessing

High-density EEG recording was acquired using a QuickAmp amplifier (Brain Products, Inc.) with 60 Ag–AgCl electrodes at standard equidistant scalp positions. The reference electrode was located at Fpz and all electrode impedances were set to <5 kΩ during recording. The data were recorded with a sampling rate of 500 Hz and later (offline) down-sampled to 256 Hz. After recording, an IIR band- pass filter ranging from 0.5 to 20 Hz (with a slope of 48 db/oct each) and a notch at 50 Hz were applied. A manual inspection of the data was performed to remove technical artifacts. Recurring artifacts caused by eye movements were removed by using the Gratton & Coles algorithm as implemented in BrainVision Analyzer II. Next, single trial segments were formed for the SC condition. The segments were stimulus-locked to the onset of the STOP+CHANGE stimulus, which was set to time point zero. Furthermore, we only included trials with correct responses (i.e. correct responses to the CHANGE stimulus before the onset of the speedup-sign and no premature or incorrect button presses either before or after the CHANGE stimulus). Each segment started 2000 ms before the onset of the STOP+CHANGE stimulus and ended 2000 thereafter, resulting in an overall segment length of 4000 ms. An automated artefact rejection excluded all segments which met one or more of the following exclusion criteria: amplitudes below −100 μV or above 100 μV, value differences of more than 200 μV in a 200 ms interval, value differences of less than 0.5 μV in a 100 ms interval. Next, a baseline correction was set to the time window from −900 till −700 ms to obtain a pre-stimulus baseline which was mostly unaffected by the GO stimulus presentation. Based on these pre-processed segments, we applied two different kinds of analyses to quantify ERP amplitudes and to identify the underlying independent components.

### ERP quantification

Based on the obtained EEG data, we analyzed ERPs to identify and dissociate cognitive sub-processes we hypothesized to be involved in multimodal information integration. For the analysis of ERPs, we separately averaged the single trial segments formed during pre-processing for each task version. Based thereon, we quantified the amplitudes of the occipital (i.e. visual) P1 and N1 at electrodes P7 and P8 as well as the amplitude of the fronto-central P3 at electrode Cz. These electrodes were chosen on the basis of visual inspection of the ERP scalp topography as well as previous studies using different variations of the Stop-Change paradigm^5, 14^. Based on grand averages of each combination of task version and condition, we chose 20 ms intervals which were optimal for the quantification of each peak (see Table 3). Within these intervals, amplitudes were averaged at the single subject level to obtain a value for the peak amplitude of each ERP.

**Table 3:** ERP quantification.

Task	vis P1 - P7/8	vis N1 - P7/8	P3 - Cz
VV	120–140	205–225	315–335
AV	120–140	205–225	230–250
TV	120–140	205–225	210–230

Time intervals used for ERP quantification in the SC condition (in ms after the STOP+CHANGE stimulus onset). VV = visual-visual task version, AV = audio-visual task version, TV = tactile-visual task version.

### Group level data decomposition using independent components

Subsequently, we decomposed the ERP signal obtained via preprocessing in an exploratory approach to identify distinct anatomical sites associated with the observed effects. To decompose the EEG data into its constituent components, a group-level second-order blind identification (SOBI) on the multi-subject EEG data was set up^16, 19^ based on the segments formed during pre-processing. This procedure estimates sources expressed concurrently across subjects, thereby naturally providing a generalization of the component structure beyond the individual. An adjusted number of trials was randomly selected from each subject’s available data after the preprocessing of the EEG data. Of note, the number of trials has to be the same for each subject in order to compute the group-level decomposition, which relies on a vertical concatenation of the single-subject data into a single matrix. In addition, the number of trials per condition was adjusted such that the average noise level of the condition-specific single-subject ERPs, computed as root mean squares of the baseline periods, did not differ between the conditions. These constraints led to a selection of 120 trials (40 trials per task version). Group-level SOBI relies on a number of consecutive processing steps. In short, each single-subject dataset first undergoes an individual principal component analysis (PCA), thereby extracting most relevant and orthogonal time courses. These first-level principal components then are used as variables in a second, group-level PCA, now estimating most-relevant and orthogonal principal component time courses capturing activity patterns correlated across subjects. Ultimately, these undergo SOBI that finally computes the component time courses. SOBI parameters were set to a lag of 50, thus a joint diagonalization of 50 covariance matrices was computed. Importantly, this nested procedure is capable of capturing some of the topographical variability we usually see with EEG events found in each of the single-subject datasets of a multi-subject study. Please refer to refs 19 and 16 for a more detailed description of the algorithm. A total of ten components were extracted. The inspection of the single-subject PCAs revealed that on average, ten components explain about 95% of the variance of the scalp EEG data. Correspondingly, at each level of this analysis, including single-subject PCAs, group PCA, and group SOBI, ten components were extracted. The ten resulting group components are characterized by their topographies and time courses, and naturally lead to a characterization of the latent data structure at the group level. To statistically analyze the data at the component level, subject-specific components (corresponding to the group-level components) were reconstructed by multiplying the original EEG data with the unmixing matrix as estimated in the previous data decomposition^16^. Subject-specific component time-courses were z-transformed to compensate for differences in scaling while preserving variations across conditions. Group components as well as the subject-specific mixing and demixing matrices were computed using self-written MATLAB scripts that followed the procedures described in refs 19 and 16. We then quantified the 20 ms time intervals yielding task version differences in the ERP analyses (i.e. P1, N1, and P3; see Table 3 and results section) for all 10 ICs. Those were then separately analyzed using the within-subjects factor task version. In addition, brain sources were computed for selected group ICs that best corresponded to the EEG effects of interest. Equivalent current dipole locations were estimated according to the variational Bayes approach as implemented in SPM12 (www.fil.ion.ucl.ac.uk/spm). Head compartment meshes were generated using the template T1-image provided with SPM12. Electrode locations were fitted based on a standard label matching procedure. A forward model was set up based on the co-registration of the head compartment meshes and three spherical shells. The source space was confined to the inner brain compartment as computed from the T1-weighted template image. Dipoles were reconstructed for a time window of 20 ms centered at a component’s peak activity, and either a single or two symmetric dipoles were fitted to identify the hot spots of neural activity to support the interpretation of ICs.

### Statistics

Separate repeated-measures ANOVAs were used to analyze behavioral, ERP, an IC data in a three-step data-guided fashion. This means that only conditions which yielded significant behavioral results were investigated during ERP analyses. Likewise, only the peaks yielding significant ERP differences were further investigated using the SOBI signal decomposition. All analyses used the within-subjects factor “task version” (VV vs. AV vs. TV). All reported values underwent Greenhouse-Geisser correction and post-hoc tests were Bonferroni-corrected, whenever necessary. For all descriptive statistics, the standard error of the mean (SEM) is given as a measure of variability.
